# Targeted Delivery of siRNA Therapeutics to Malignant Tumors

**DOI:** 10.1155/2017/6971297

**Published:** 2017-11-09

**Authors:** Qixin Leng, Martin C. Woodle, A. James Mixson

**Affiliations:** ^1^Department of Pathology, University of Maryland School of Medicine, 10 S. Pine St., Baltimore, MD 21201, USA; ^2^Aparna Biosciences Corporation, 9119 Gaither Rd., Gaithersburg, MD 20877, USA

## Abstract

Over the past 20 years, a diverse group of ligands targeting surface biomarkers or receptors has been identified with several investigated to target siRNA to tumors. Many approaches to developing tumor-homing peptides, RNA and DNA aptamers, and single-chain variable fragment antibodies by using phage display,* in vitro* evolution, and recombinant antibody methods could not have been imagined by researchers in the 1980s. Despite these many scientific advances, there is no reason to expect that the ligand field will not continue to evolve. From development of ligands based on novel or existing biomarkers to linking ligands to drugs and gene and antisense delivery systems, several fields have coalesced to facilitate ligand-directed siRNA therapeutics. In this review, we discuss the major categories of ligand-targeted siRNA therapeutics for tumors, as well as the different strategies to identify new ligands.

## 1. Introduction

In tumor-bearing mice, nanoparticles (NPs) greater than 10 nm in diameter have a proclivity to accumulate in the tumor. Accumulation of these NPs in tumors, however, is modest and only about 20 to 40% greater than its accumulation by normal tissues [1]. The preferential uptake of NPs by tumors is due to the enhanced permeation and retention (EPR) effect. The EPR effect is thought to result from a combination of leakiness of tumor blood vessels resulting in flux of NPs from the blood into the tumor tissue and reduced numbers of lymphatic vessels in tumors associated with decreased drainage of NPs and occurs despite a higher interstitial pressure within the tumor. Notably, several rarely used therapies, such as nitroglycerin, may enhance the EPR effect and augment accumulation of NPs within tumors [2, 3]. When NPs have diameters less than ~10 nm, they are rapidly secreted by the kidneys and the effect of EPR is greatly reduced [4]. Moreover, NPs with very short half-life and/or with their nonspecific binding may accumulate within the tumor to a greater extent, if the EPR effect is enhanced by pegylation of particles. By prolonging blood circulation (plasma half-life) of the NP and reducing nonspecific binding, pegylation may also enable accumulation of ligand-nanoparticle conjugates in tumors above the EPR effect.

Building on accumulation of NPs in tumors from the EPR effect, researchers have sought to increase their tumor delivery by coating the particles with tumor-localizing ligands. The mechanism by which ligands increase the antitumor efficacy of their cargo (in our case siRNA) is somewhat controversial. Most investigators have determined that increased efficacy of targeted ligand-siRNA NPs is due to enhanced binding to the tumor surface marker and accumulation of NPs in the tumor compared to that in nontargeted tissues. Some investigators, however, have found that accumulation of targeted and nontargeted NPs within tumors was similar and found that increased efficacy of the targeted NP was due to enhanced receptor-mediated endocytosis and increased intracellular localization of the siRNA therapeutic [5]. Most likely, both mechanisms have important roles in ligand- targeted therapy, improving efficacy, and depend on the delivery vehicle, the target of the ligand, and strategy used in making the ligand (i.e., aptamer, peptide, or antibody).

**Figure 1:**
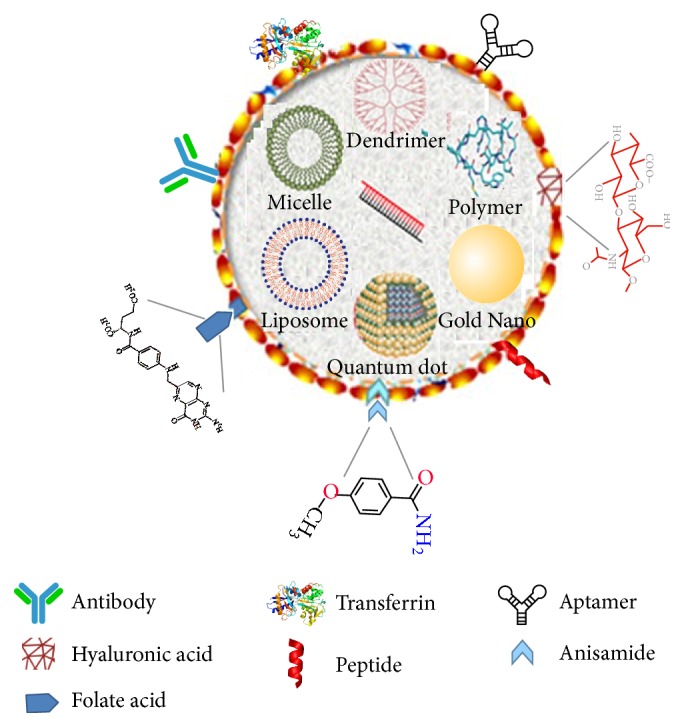
Schematic overview of the different ligands and core particles that target tumors. An array of core particles and ligands has been used to carry siRNA which inhibit oncogenes or induce apoptosis of tumor cells.

In this review, we describe various strategies that have been developed for ligand-siRNA therapeutics to increase their selectivity toward tumors (Figure 1). “Decorating” the NP with the ligand together with PEG shell, however, does not adequately describe how ligand molecules may affect stability of the core particle. As investigators have reported, ligand molecules and their specific linkages to the NP may significantly influence release of siRNA and their efficacy [6].

In addition, the efficacy (or lack thereof) of the siRNA-NP may interfere with the independent evaluation of ligand-directed therapies. It is our goal in this review not only to cover the array of ligand-targeted siRNA NPs, but also to indicate possible flaws in the particular study and alert the reader to the potential of the ligand, independent of the efficacy of the NP. This determination will be particularly important in cases in which there has been reduced antitumor efficacy with the nanoparticle.

## 2. Ligands Targeting Tumor Cells and Vessels

**Figure 2:**
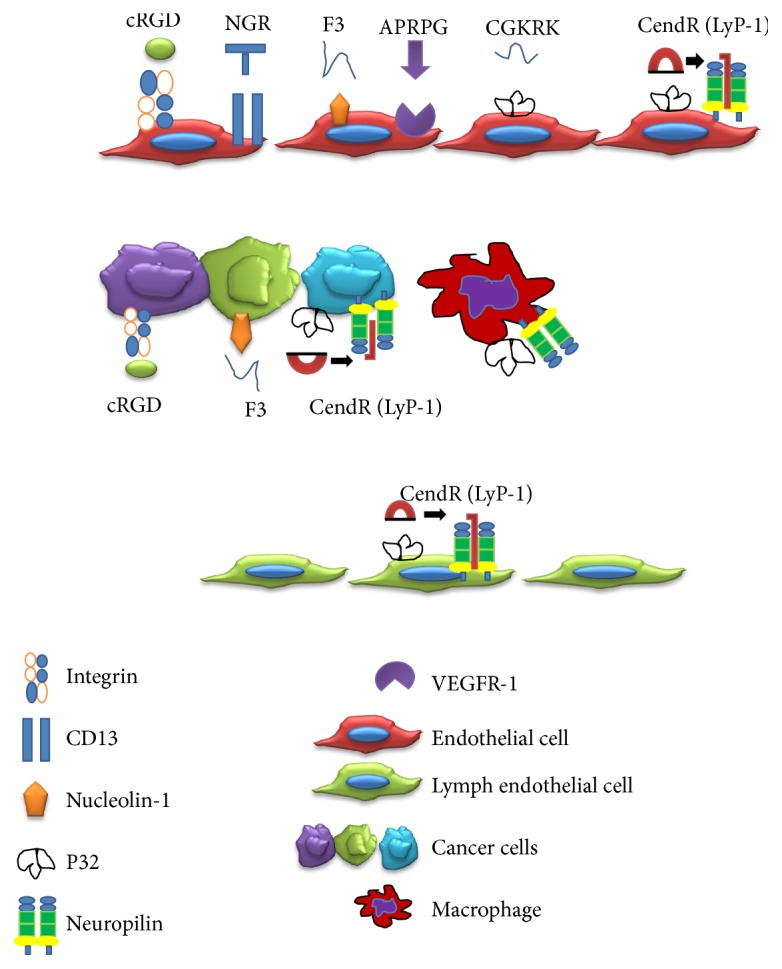
Peptide ligands targeting tumor endothelial cells and tumor cells. Ligands and their receptors are shown with associated cells.

Ligands targeting tumor cells and their angiogenic vessels have primarily been peptides isolated by the phage display method (Table 1) (Figure 2) [7–15] (see review by [16]). Since tumor cells and angiogenic blood vessels often have similar cell surface receptors, ligands can have dual targeting capabilities for both tumor vasculature and tumor cells. When this is the case, siRNA therapeutic agents that have an inhibitory effect on both cells may be preferred for use with these targeting ligands, as recently reported with a miRNA mimetic [17].

Of note, a few promising targeting peptides discussed in this section have not been tested for their efficacy with siRNA therapy although they have shown their ability to target tumors with cytotoxic peptides and chemotherapeutic drugs. Moreover, except for cRGD-targeted therapies, the remaining siRNA studies are limited and additional studies, particularly with CendR and NGR targeting peptides, are required to confirm the encouraging* in vivo* results.

### 2.1. Cyclic(c) RGD

cRGD, isolated from an* in vivo* tumor phage selection display, was found to bind to *α*v*β*3 and *α*v*β*5 integrins. These two integrins are highly expressed on angiogenic endothelial cells of tumors as well as on a number of tumor cells (about 10–15%). Interestingly, *α*_v_*β*3 and *α*v*β*5 both have important roles in tumor angiogenesis, yet they control different aspects of angiogenesis. Whereas *α*v*β*3 has an important role in the bFGF-mediated angiogenic pathway, *α*v*β*5 has a more critical role with the VEGF-mediated pathway [18]. The functions of these integrins have not been clearly defined in tumor cells, yet they do modulate apoptosis.

The cRGD ligand has been extensively used for targeted therapy against an array of cancer models. cRGD binds to integrins at least 100-fold more tightly than linear RGD [19]. Although the initial cRGD required oxidation of cysteines to achieve its cyclic form (A[CRGDMFGC]A), the use of D-phenylalanine enabled cyclization of the peptide (cRGDfV) without the unwanted cys-cys reactions [19, 20]. An important paper demonstrating the efficacy of cRGD therapy was published by Hood and colleagues in 2002 [21]. This plasmid-based approach with cRGD liposomal carriers of dominant-negative mutants of Raf-1 not only inhibited tumor xenografts, but also resulted in their regression. This study provided the stimulus for siRNA therapy targeting* RAF-1*,* VEGF*,* VEGFR2*,* ERG*,* FAK*, and* PLXDC1* and several other oncogenes [22–28].

Antiangiogenic therapy with siRNAs was first demonstrated with cRGD-containing PEI siRNA-NP targeting* VEGF* (si*VEGF*) to inhibit the growth of N2A murine neuroblastomas [22]. Coadministered free excess cyclic RGD markedly decreased the efficacy of the targeted therapy, demonstrating the specificity of cRGD-NP. Later, a cRGD functionalized chitosan NP targeting* PLXDC1* (upregulated in the tumor vasculature) inhibited ovarian tumor growth by about 90%; the cRGD-NP was about 60% more effective than the untargeted NP [23]. Since the A2780 tumor cell did not express *α*v*β*3 integrins, the authors concluded that the efficacy of the therapy was its direct effect on the endothelial cells. In contrast, Chou et al. utilized an *α*v*β*3-expressing melanoma cell line in their murine model and directed their therapy toward genes expressed in the tumor cells [24, 29]. Chou and colleagues reduced luciferase expression by more than 80% in MDA-MB-435 tumor xenografts with their RGD-biodegradable NP carrying luciferase siRNA. Moreover, when the Raf-1 siRNA RGD-NP targets human* RAF-1* in the melanoma cells, but not the mouse* Raf-1* (in blood vessels), the tumor was inhibited by more than 60% [29]. When cRGD-PEG-NPs were compared with PEG-NPs for reduction in tumor luciferase expression, the targeting cRGD moiety was responsible for 50% of reduction of their target gene. Christie et al. targeted both VEGF and VEGFR2 with their cRGD micelle-siRNA particle, which was stabilized with 2-iminothiolane; compared to untreated tumors, these particles reduced tumor growth by more than 80% [27]. Christie et al. noted that the combination of* VEGFR2* and* VEGF* siRNAs was more effective than administration of VEGF siRNA alone. Targeting VEGFR2 located on endothelial cells by the cRGD-NP is likely to be very effective because of its ready accessibility, whereas targeting secreted VEGF from both tumor and endothelial cells may be less effective, due to reduced penetration of the tumor by the NP.

### 2.2. APRPG

APRPG peptide, first found from a phage-displayed peptide library in 2002, is an angiogenic vessel-homing peptide [11], which binds to VEGFR-1 [30]. There have been several studies with APRPG-conjugated NPs including investigations that incorporated antiangiogenic or chemotherapeutic agents within targeted liposomes. Doxorubicin, TNP-470, and the tyrosine-kinase VEGFR2 inhibitor-SU1498 which were encapsulated within APRPG-liposomes, showed significant antitumor activity* in vivo, *including colon and ovarian cancers [31–33]. Compared to the nontargeted NP, the targeted NP showed greater efficacy at downregulating its target and in most cases, they showed significantly greater antitumor activity.

In addition to chemotherapy and tyrosine-kinase inhibitors, siRNA [34, 35] and miRNA [36] have been incorporated within APRPG-NPs. Although APRPG primarily targets angiogenic vessels, Lu et al. interestingly found that APRPG targeted PEI in complex with* VEGF* siRNA inhibited the growth of breast cancer MCF-7 cells* in vitro* more effectively than untargeted PEI-polyplexes [34]. Inhibition of tumor growth, however, with the targeted PEI complex was no greater than the nonligand NP. Nonetheless, the targeted PEI polyplex reduced intratumoral VEGF mRNA and VEGF significantly more than the nontargeted polyplex. In a lung tumor model in which wild-type PTEN was expressed, liposomes which were decorated with APRPG and carried si-*mTOR* markedly reduced the tumor burden compared to the phosphate-buffered saline-treated group [35]. Although the targeted ligand NP accumulated in the lung tumors significantly more than nontargeted NPs, inhibition of the tumor was similar to the targeted and untargeted therapies. The authors speculated that the discrepancy between accumulation and the antitumor efficacy may be due to differences in the distribution of the liposomes within the tumor. Whereas the targeted NP may be primarily located in the tumor vasculature, the nontargeted NPs may be found throughout the tumor interstitium.

### 2.3. NGR

Cyclic NGR (i.e., CNGRCVSGCAGRC) is a ligand for CD13 [8], which is upregulated primarily in the tumor vasculature and in a few cancers such as fibrosarcomas. Although the cyclic NGR binds with higher affinity than linear NGR, and drug conjugates with the cyclic peptide have 10-fold greater antitumor efficacy, the linear NGR has been the preferred ligand on the surface of nucleic acid carriers because it avoids cysteine bridges that may occur between different carrier molecules. In addition, biodistribution and targeting with linear NGR conjugated to liposome surfaces have been reported to be quite good, probably because of the multivalence presentation of the ligand on the NP. Recently, Negussie and colleagues developed a cyclic NGR (cKNGRE) without disulfide bonds, which had about 3.5-fold greater affinity (with and without liposomes) toward CD13^+^ cells compared to the linear NGR (KNGRG) [37]. Furthermore, the linear and cyclic peptides when conjugated to liposomes both had a 10-fold greater affinity for CD13^+^ cells compared to their respective free peptides. With an estimated ~4200 peptides per liposome, it is not clear nor has it demonstrated that the cyclic NGR conjugated to liposomes would improve their biodistribution or antitumor activity compared to noncyclic NGR liposomes. With NPs (or proteins) containing a limited number of NGR peptides on their surface, the cyclic peptide may have a greater role in enhancing binding and selectivity.

To the best of our knowledge, there has only been a single NP-siRNA study that used NGR. In this report, the linear NGR (GNGRGGVRSSSRTPSDKY) was attached to the PEG on a liposomal polycationic DNA (LPD) carrier of siRNA. The NP delivered si-cMYC to the cytoplasm and downregulated the target gene in the CD13^+^ HT 1080 cells but not CD13^−^ HT-29 cells* in vitro *[38]. Notably, the NGR si-*cMYC* NP reduced tumor size of HT-1080 xenografts by about 30% more than untargeted NP. When doxorubicin was in complex with DNA, the targeted NP containing doxorubicin/DNA and si-*cMYC* reduced the size of tumors by a further 40% compared to the particle containing si-*cMYC* alone [38].

### 2.4. F3 Peptide

The F3 peptide (KDEPQRRSARLSAKPAPPKPEPKPKKAPAKK), a 31-amino acid fragment of HMGN2 protein, binds to nucleolin which is on the cell surface of tumor and angiogenic endothelial cells [39]. After the F3 peptide binds to nucleolin at the cell surface, they are rapidly internalized and transported to the nucleus [40]. Compared to untargeted liposomes, a F3 peptide-modified liposomal carrier of siRNA enhanced internalization by 10-fold in both breast cancer cells and angiogenic blood endothelial cells and this uptake correlated with more effective gene silencing [41]. Moreover, these investigators showed that the F3 ligand conjugated to liposomes containing si*PLK1* (polo-like kinase-1) reduced cell viability by 40% and sensitized the PC3 prostate cancer cells to paclitaxel (IC_50_ reduced by about 45%) [42]. Similar synergistic results with an apoptotic siRNA (AllStars Death-siRNA, Qiagen) and paclitaxel were observed for ovarian cancer cells with a F3 targeted multifunctional NP [43]. Numata and colleagues were the only group to demonstrate the efficacy of F3-targeted NPs to tumors* in vivo*, using a plasmid-based gene delivery system [44]. Compared to the vascular homing (CGKRK) particles, the F3-targeted silk particles enhanced luciferase expression in the two malignant cells (MDA-MB-435 and MDA-MB-231)* in vitro. *Neither targeted peptide NPs (CGKRK or F3) resulted in measurable luciferase activity in a nonmalignant cell. For the* in vivo* study, the F3-nanoparticles (injected iv twice a week) markedly enhanced luciferase expression after the first week in MDA-MB-231 derived xenografts but F3-targeted particles were not compared with non-F3 particles as carriers of luciferase. Notably, utility of the F3 peptide conjugated to siRNA NPs* in vivo* has not yet been examined.

### 2.5. CGKRK

CGKRK targets primarily the angiogenic vessels of tumors. Similar to LyP-1 peptide (see below, Section 2.6), it binds with high affinity to the p32 receptor which aids in the transport of the conjugate or NP into the cytosol of the angiogenic vessels. Unlike LyP-1, the CGKRK does not contain a CendR motif with tumor-penetrating properties, and consequently, NPs containing CGKRK primarily targets endothelial cells of the tumor. Based on targeting of angiogenic vessels, the CGKRK targeted a dimer of the proapoptotic peptide, klaklak (D-amino acids) to brain glioma xenografts, resulting in its enhanced accumulation in the tumor vasculature and prolonged survival of mice [45]. To date, there have not been any reports of siRNA NPs with the CGKRK targeting ligand* in vitro* or* in vivo*.

### 2.6. Tumor-Penetrating Peptides with the CendR Motif

**Figure 3:**
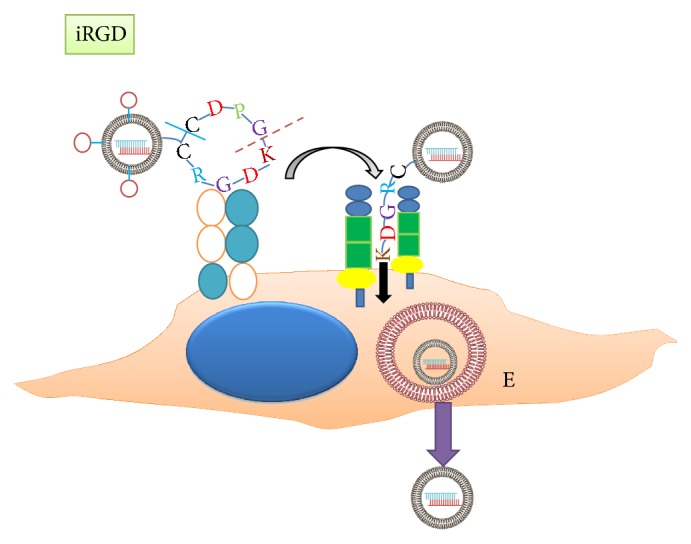
Proposed mechanism of CendR tumor-peptides to transport NPs into tumor matrix. After a CendR peptide such as iRGD binds to av integrins, furin-like enzymes cleave the cyclic peptide (dash line) on the carboxyl side of the lysine group. With reduction of the cystine linkage (solid line), the peptide, KDGR, binds to the neuropilin receptor and activates the transcytosis pathway. The peptide together with the NP is then endocytosed and transported through the endothelial cell to the tumor milieu. E, endosome.

In contrast to the tumor-homing peptides discussed above (i.e., cRGD and NGR), tumor-penetrating peptides and their associated NPs penetrate deeply and pervasively throughout the tumor parenchyma. There have been three tumor-penetrating peptides identified thus far: LyP-1, iRGD, and iNGR. Whereas LyP-1 and iRGD were the first two tumor-penetrating peptides to be identified and were isolated by in vivo phage display, iNGR was the result of de novo synthesis gained from knowledge of the other two peptides [7, 12, 14, 15, 46, 47]. Prototypical sequences of LyP-1, iRGD, and iNGR are CGNKRTRGC, CRGDKGPDC, CRNGRGPDC, respectively, with the CendR motif underlined. For extravasation of NPs into tumors, the peptide requires two motifs, one for tumor homing and the other for tumor penetration. With the exception of LyP-1, the peptides must be cyclic for optimal binding and specificity for tumor homing. Moreover, for tumor homing of the NP, the LyP-1 peptide binds to p32 receptors on tumor cells, tumor blood vessels and lymphatics, and tumor macrophages, whereas the iRGD and iNGR peptides initially bind to *α*_v_*β*3 integrins and CD13 receptors, respectively, on the angiogenic vessels of tumors. After the tumor-specific components of the penetrating peptide bind to the specific receptor in the tumor, proteolytic degradation exposes the CendR sequence (R/KXXR/K-OH, where R/K represents either arginine or lysine and X represents any amino acid; see underlined sequences above). Exposure of the cryptic CendR activates the neuropilin-1 and neuropilin-2 pathways, enhancing extravasation of the NP into the tumor. At least part of the extravasation of the NP is thought to be due to transcytosis [48] (Figure 3).

Although several studies show that tumor-penetrating peptides improve antitumor efficacy of drugs or NPs incorporating drugs [12, 47, 49, 50], there have been few studies using tumor-penetrating peptides with siRNA (or shRNA) nanoparticles [10, 51]. Ren and colleagues demonstrated that LyP-1 siRNA NPs accumulated 3-fold more in tumors compared to untargeted NPs [10]. Moreover, location of the targeted NP within the tumor was extravascular in contrast to the intravascular location of the untargeted NP. With a LyP-1 containing NP carrying an si*IDF* (inhibitor of DNA binding 4), ovarian cancer xenografts were reduced by nearly 80% compared to tumors treated with the si*GFP* control group. A similar study was done with iRGD-NPs carrying both paclitaxel and a plasmid-based shRNA targeting survivin [51]. The size of the tumors treated with iRGD targeted NPs remained unchanged during the course of therapy (~280 mm^3^), whereas size of tumors treated with untargeted NP or saline grew to about 900 and 2200 mm^3^, respectively.

In contrast to cRGD peptides-NPs that do not contain cysteines, iRGD-NPs with cysteines may have reduced stability in solution when reconstituted due to intermolecular bridge formation between NPs from reduced cysteines. The tendency of reduced cysteines to form these side reactions may limit the clinical utility of cyclic iRGD/iNGR conjugated to NPs. Importantly, not only administration of iRGD-NPs but also coadministration of iRGD and NPs significantly enhanced tumor penetration and antitumor efficacy of the NP [14].

## 3. Targeting Tumor Cells

The diverse approaches and ligands to target tumors cells have indeed been numerous. Whereas ligands targeting angiogenic vessels were peptides as discussed previously, the ligands targeting markers/receptors on tumor cells have ranged from single-chain variable fragment antibodies to RNA aptamers and from low molecular weight ligands such as folate to high molecular weight ligands such as transferrin and hyaluronic acid (Table 2). The ligands in this section primarily target tumor cells although they may target other tumor-associated cells to a lesser extent.

### 3.1. High MW Endogenous Ligands 

#### 3.1.1. Transferrin

To deliver the growth mediator iron intracellularly, the transferrin receptor (TfR) is markedly upregulated in many tumor cells, as much as severalfold higher than in normal cells [52]. When iron binds to its receptor, they are endocytosed and the iron is released in the acidic endosomal compartment and the receptor is recycled to the surface [53]. Transferrin has been evaluated extensively as a ligand to enhance the delivery of drugs and NPs to tumors [5, 54–58]. The most extensively studied transferrin-targeted siRNA targeting tumors was based on the cyclodextrin carrier. Marked suppression of the oncogene (M2 subunit of ribonucleotide reductase) [59] or exogenous marker (luciferase) [60] was reported with intravenous delivery of the transferrin-NP. Thus far, this has been the only targeted ligand-siRNA NP (CALAA-01) tested in a cancer clinical trial (this therapy was discontinued after phase 1 due in part to adverse clinical events) [61]. Other investigators have found that transferrin coupled to NP enhanced the specificity and potency of the NP within the tumor [56]. For example, Liu et al. found that transferrin-PEI-plasmid shRNA (targeting* HIF-α*) accumulated in a time-dependent manner in tumors expressing high levels of the TfR [56], reducing the growth rate by 8-fold compared to control groups. Interestingly, biodistribution studies did not detect a difference in accumulation within the tumor between untargeted and transferrin-containing NPs, despite their having marked differences in their antitumor efficacies [5]. The authors indicated that transferrin was essential for delivery of the NP and siRNA intracellularly. Studies with DNAzymes carried by the transferrin-cyclodextrin also support the notion that internalization within the tumor cell and not accumulation and localization within the tumor is the important factor [55].

Notably, the cyclodextrin studies from Davis group also underscored that the method to form the targeted nanoparticle was important [62]. Whereas grafting of PEG-ligand onto preformed polyplexes may augment transfection of PEI-polyplexes, polyplexes formed with the low molecular weight cyclodextrin may be destabilized by this approach, particularly in the presence of salt. When cyclodextrin, nucleic acids, and adamantine-PEG-ligand were mixed together, polyplexes were stable in different salt concentrations and maintained their ability to target cells. Although this approach was initially used for cyclodextrin DNA polyplexes, this strategy was later used to form stable targeted cyclodextrin-siRNA polyplexes. In many cases, optimal targeting is dependent on the method used to form the nanoparticle.

#### 3.1.2. Hyaluronic Acid

Hyaluronic acid (HA), a negatively charged natural polymer, has recently been investigated as a targeting agent for drug delivery systems. HA binds to the surface CD44 receptors, which are overexpressed in primary and metastatic tumor cells, and consequently, several studies have utilized HA-coated NPs in which siRNA has been incorporated to enhance antitumor efficacy. After determining that HA-grafted liposomal carriers of si*PLK1* reduced its target gene expression* in vitro* by about 90%, Cohen and colleagues investigated the efficacy of these HA-grafted liposomes* in vivo *[63]. In a glioblastoma orthotopic mouse model, the HA-grafted liposomal carrier of si*PLK1* administered by local injections markedly prolonged mouse survival. Compared to the untreated and si*Luc* control groups in which all mice had died prior to day 40, six of the ten mice in the HA-liposomal-si*PLK1* group survived to day 95. In addition to liposomes, a chitosan-siRNA-NP, which was coated with HA, showed enhanced uptake, particularly in cells expressing high levels of CD44 [64].

Other targeting ligands have been added to the surface of HA-coated NPs to further enhance delivery to tumors of nucleic acids, although this approach has not been used for siRNA therapeutics yet. For example, Yu's group determined that transferrin and HA-coated liposomes enhanced plasmid-based gene expression (EGFP) to A549 lung adenocarcinoma xenografts by about 40% compared to either single-ligand liposomal NP [65]. Similarly, micelles coated with HA and folate increased accumulation of paclitaxel by about 42% in MCF7 xenografts compared to HA-only coated micelles [66]. On the bases of these studies, the approach of dual-ligand targeting for siRNA delivery has a great deal of promise for hyaluronic acid and the other ligands discussed in this review.

Besides coating preform NPs with HA, alternative strategies have been used to conjugate (or complex) cationic polymers or cholesterol with HA. Discrete and limited conjugation of cationic polymers, such as PEI, polyarginine, and polylysine, with HA has been done to form positive and negative surfaces [67, 68]. Whereas the positive surface of the modified HA binds to the siRNA, the negatively charged HA coating the NP can bind to CD44-positive tumor cells. Choi and colleagues have also conjugated cholesterol with HA to form micelle-like NPs. To incorporate siRNA within these NPs, Choi et al. utilized the viral protein, 2b, which specifically binds siRNA in a pH-dependent manner [69]. Although non-HA micelles were not used as controls, the HA-cholesterol micelles were more effective in silencing red-fluorescent protein (about 80%)* in vitro* compared to Lipofectamine 2000 (about 64%).

#### 3.1.3. ApoA1

Apo-A1-HDL (rHDL) particles bind tightly to the scavenger receptor class B-type 1 (SR-B1), which is found predominantly on the surface of hepatocytes. Not surprisingly, rHDL particles incorporating cholesterol siRNA conjugates have been injected intravenously at low dosages (≤2 mg/kg) to silence genes in a hepatitis B mouse model, resulting in a marked decrease of the hepatitis surface antigens [70]. Moreover, it has been recognized that the SR-B1 receptor is upregulated in several tumors, ranging from hepatocellular to breast cancers. With an siRNA conjugate encapsulated within rHDL-coated particles, Ding et al. demonstrated the utility of this delivery method by targeting an oncogene (*VEGF*, Pokemon, or* BCL-2*) with tumor uptake and tumor inhibitory studies [71, 72]. Accumulation of the rHDL particle was significantly higher in MCF 7 xenografts (5.5-fold higher) which expressed increased levels of SR-B1 compared to the HT1080 tumor xenografts [72]. Moreover, mice with MCF-7 tumors treated with rHDL-si*VEGF* had a further 70% reduction in their size compared to nontargeted NPs or to untreated mice. Concomitant with the decrease in tumor size, marked reduction in VEGF mRNA levels and numbers of microvessels in the tumor were also observed in the rHDL-siVEGF-treated group compared to untargeted NPs and untreated groups.

### 3.2. Aptamer

**Figure 4:**
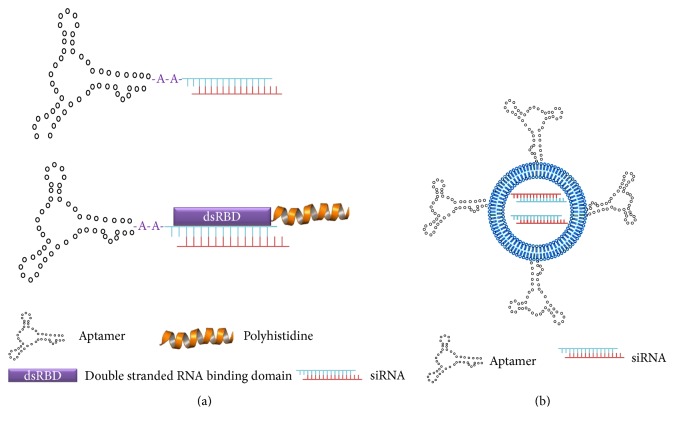
Aptamer mediated delivery of siRNA. (a) Aptamers and siRNAs have been conjugated with one another to form a chimera (upper). To enhance lysis of endosomes and minimize formation of polyplexes, aptamer-siRNA conjugates have been complexed to double stranded DNA domain- (DSD-) polyhistidine conjugates (lower). Upon entry into acidic endosomes, the polyhistidine component becomes protonated which aids in the lysis of endosomes. (b) Alternatively, aptamers have been conjugated to the surface of core particles (i.e., liposomes, polyplexes) that have incorporated siRNA.

Aptamers are small molecular weight (8–13 KDa) single-stranded RNA or DNA molecules with low nanomolar binding affinities toward their targets and with low immunogenicity (for supplementary reviews, see [73, 74]). These nucleic acid ligands are isolated from combinatorial libraries by a method known as SELEX (Systematic Evolution of Ligands by exponential enrichment). Large quantities of aptamers can readily be synthesized and degradation of aptamers by nucleases may be avoided through chemical modification. By binding with high affinity to target molecules, investigators have found a number of biological applications for aptamers, including target validation, inhibitors of receptors or enzymes, carriers for nucleic acids and chemotherapy agents, and ligands conjugated to NPs. Recently, clinical trials with aptamers alone as antitumor agents have been initiated [75]. For siRNA studies targeting tumors, we will provide an overview on aptamer-siRNA chimeras and aptamer-siRNA NPs (Figure 4).

#### 3.2.1. Aptamer-siRNA Conjugates

Initially most investigators focused on aptamers-siRNA conjugates (Figure 4(a), upper). With aptamers-siRNA conjugates, aptamers serve two purposes: one as a carrier of siRNA and the other as a ligand targeting a tumor-associated antigen. With these conjugates, the aptamer and siRNA could be joined by either a linker or directly as a chimera.

Chu et al. developed a first-generation aptamer that targeted prostate-specific membrane antigen (PSMA), which is highly expressed on LNCaP prostate cancer cells [76]. Through a streptavidin-biotin linkage, a laminin A/C siRNA was linked to the aptamers with the conjugate reducing its target gene levels by about 70%. Moreover, LNCaP tumor xenografts regressed by 2.21-fold from days 6 to 21 after ten intratumoral injections (every other day) of the anti-PSMA aptamer-si*PLK* chimera; in contrast, the tumors in the control group increased by 3.63-fold [77]. The chimera had no effect on the size of PC3 prostate tumors, which do not express PSMA.

Building on these findings, Dassie et al. made several modifications to a PSMA-si*PLK* chimera that resulted in 22Rv tumor regression when the chimera was administered systemically (one injection per day for 10 days) [78]. Indeed, 70% of the tumors regressed completely from a pretreatment size of about 400 mm^3^. This is indeed quite remarkable, although it has been noted that high dosages of chimera were used, suggesting that significant amounts of the chimera were entrapped in the lysosomal pathway. In contrast to the 22Rv tumors, the chimera had no effect on the PC3 tumors. Particularly relevant modifications of the chimera that augmented* in vivo* activity included pegylation to increase its half-life, addition of 2′-fluoropyrimidines on the passenger strand of the siRNA to decrease nuclease activity, 2 nucleotide 3′ overheads, and swapping the position of the guide and passenger strands. These modifications significantly improved the ability of the chimera to reduce tumor size and/or to decrease PLK1 expression. For instance, pegylation of the chimera silenced PLK1 mRNA in the tumor by about 45% 5 days after a single injection, whereas the nonpegylated form had no effect. Consistent with this finding, pegylation of the chimera had an increased half-life to more than 30 hours, whereas the nonpegylated chimera had a half-life of less than 35 minutes. A similar strategy but with different targets for the siRNA and aptamer was used by Subramanian et al. [79]. By targeting EpCAM with the aptamer as well as with a siRNA of* EpCAM*, the systemically delivered chimera resulted in the regression of tumor xenografts derived from MCF-7.

Since most aptamer-siRNA chimeras are degraded in the lysosomal pathway, efforts have been made to increase endosomal lysis, enabling the escape of the siRNA intracellularly. One such strategy is a fusion PSMA aptamer-dsRNA binding domain-polyhistidine dual block protein, in which 1 or 2 siRNA docked with the binding domain and the polyhistidine enabled endosomal lysis (Figure 4(a), lower) [80]. Addition of an 18-mer of polyhistidine was optimal in augmenting endosomal escape and increased gene silencing (GFP) by about 5-fold in LNCaP cells. Interestingly, addition of histidine-tags of 24 or 30 amino acids inhibited siRNA binding to the RNA-binding domain. Although the authors state that these chimeras remain discrete in solution, we would be surprised if the polyhistidine tag did not form aggregates at least to some degree, based on the proclivity of histidines to interact with each other through hydrogen bonding [25].

#### 3.2.2. Aptamer-siRNA-Nanoparticles

Several aptamers, including those targeting EpCAM, TfR (CD71 or the transferrin receptor), CD30, PSMA, and a T-cell marker, have been conjugated to NPs (Figure 4(b)) [81–86]. The potential advantage of aptamer-targeting NPs is that endosomal lysis properties can be readily incorporated within the particles to release the siRNA into the cytosol, potentially a significant limitation with the aptamer-siRNA chimeras. Nevertheless, there are potential disadvantages of aptamer-targeting NPs such as greater size, limited tumor penetration, and increased complexity in their preparation compared to the aptamer alone or the aptamer-siRNA chimeras. With one exception utilizing miRNA, the aptamer-siRNA-NP has been limited to studies* in vitro*.

In 2010, a sub-10 nm aptamer conjugated to the polyamidoamine (PAMAM) dendrimer nanoplex was developed by Zhou et al. [87]. Although no RNAi cargo was incorporated, the group demonstrated that the targeted nanoplex had high affinity for a surface receptor on the T-lymphocytic lymphoma cell line. The DNA aptamer, sgc8c, which binds to the leukemia biomarker PTK7, was identified by a cell-SELEX method. Bagalkot and Gao then demonstrated that a PSMA aptamer-siRNA conjugated to PEI which coated a quantum dot significantly enhanced uptake with concomitant greater silencing of green fluorescent protein (GFP) in prostate cancer cells compared to nontargeted controls [83]. Critical to enhancement of this targeted nanoparticle was a 2-step method of preparation, thereby preserving the targeting function of the aptamer. Zhao and colleagues targeted the CD30 surface biomarker found primarily on anaplastic large cell lymphomas (ALCL) [82]. The CD30-aptamer PEI siRNA nanoplex bound selectively to Karpas 299 cells (an ALCL line), whereas this targeted nanoplex showed little binding to Jurkat cells, a cell line that does not have the CD30 surface marker. Not surprisingly, selective gene silencing for cells with C30 surface marker was demonstrated with the targeted nanoplexes. In Karpas 299 cells expressing GFP, there was a 71% reduction in those treated with the targeted nanoplex where in contrast, there was no reduction in GFP in Jurkat cells treated with nanoplexes. Similar results were observed with the targeted nanoplexes silencing the ALK gene with greater than a 60% reduction in cell number of Karpas 299 cells compared with the untreated and nontargeted control groups, whereas the targeted nanoplexes and control groups had no effect on the proliferation of non-CD30 expressing Jurkat cells. More recently, Subramanian et al. have developed a polymeric-nanoplex containing an* EpCAM* siRNA and aptamer targeting the EpCAM adhesion molecule on tumor cells [85]. The EpCAM surface protein and mRNA were efficiently downregulated by the targeted nanoplex compared to the nontargeted nanoplex. Moreover, whereas the scrambled siRNA nanoplex had negligible effect on the proliferation of the cells, the targeted nanoplex reduced cell number by about 70%.

Although aptamers have been primarily conjugated to polymeric carriers, Wilner and colleagues conjugated an anti-TfR aptamer (C2) to the SNALP liposomal carrier of siRNA [84]. Compared to the high affinity form of transferrin for its receptor, the C2 aptamer had greater affinity (see Tables 2 and 3). Both uptake and silencing activities of the targeted NPs were reduced by addition of transferrin to the medium, indicating the important role for TfR-mediated endocytosis. Moreover, the targeted and untargeted siEGFR2 NPs reduced EGFR2 mRNA by about 75% and 55%, respectively, compared to untreated HeLa cells.

While there have not been any targeted aptamer-NP studies using siRNA* in vivo*, there was an interesting report with microRNA [86]. By using a bone metastatic model of prostate cancer, Hao and colleagues demonstrated that a PSMA-functionalized atelocollagen carrier of miRNA (miR-15-A and miR-16-1) prolonged the survival of mice significantly (*p* < 0.05) (mean survival: 57 days) compared to saline-treated (mean survival: 27.2 days), targeted control miR (mean survival: 28.2 days), or untargeted miR-15-A/16-1 (mean survival: 38 days) NP groups [86]. These* in vivo* results were consistent with the* in vitro* studies in which the targeted NP had about a 4.4-fold lower IC_50_ for the LNCaP prostate cancer cells compared to the untargeted NP. The miR-15-A/miR-16-1 downregulated a number of proteins important for tumor invasion, proliferation, and survival, indicating the utility of these tumor suppressor microRNAs for targeted therapy. We expect that more* in vivo* studies will follow based on these* in vitro* and* in vivo* studies.

### 3.3. Antibody

**Figure 5:**
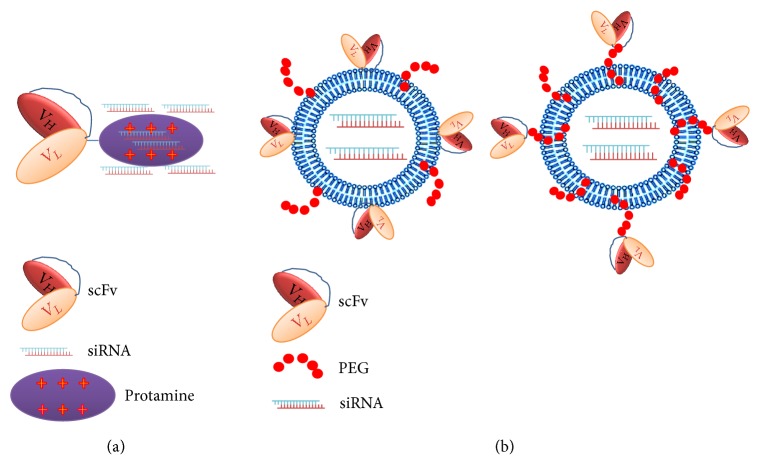
Antibody mediated delivery of siRNA. (a) siRNA can interact with the cationic proteins such as protamine that has been directly conjugated with the antibody. (b) Similar to aptamers, antibodies may be conjugated to a carrier of siRNA (liposomes, polyplexes). In addition to their direct conjugation to the membrane surface of NP, the antibody may be attached to PEG (far right). The antibody ligand includes not only the parent antibody or its Fab fragment, but as in this case, it may include the single-chain variable fragment form of the antibody.

In contrast to small molecule toxins, siRNA conjugated to antibodies have shown reduced antitumor efficacy and have not advanced to clinical trials. Whereas the efficacy of most radioisotopes- or chemotherapy-antibody conjugates is not usually reduced from enzymatic degradation by lysosomes, the siRNA component of the conjugates is susceptible to enzymatic degradation and must be delivered intact intracellularly to have a biological effect. Thus, the targeted siRNA therapy must escape from acidic endosomes before reaching the lysosomes. Moreover, antibody-toxins have been determined to be more effective against leukemia and lymphomas compared to solid tumors. Penetration of solid tumors may not be effective with the large molecular weight antibodies, and of course, penetration of the antibodies conjugated to NPs could present an even greater challenge. As a result, the trend in the siRNA field is to use scFv or Fab fragments, because of their small size and their ability to deliver silencing activity with their siRNA similar to the much larger parent antibodies. When conjugated directly to a cationic peptide or to NP, the smaller MW Fab antibody fragments or single-chain variable fragment antibodies have a much higher likelihood of better penetration in solid tumors. Single-chain variable fragment antibodies are about 12–15 kDa in size, smaller than the Fab fragments which are 50 kDa, and significantly smaller than the parent antibodies of 150 to 160 kDa. Thus, scFv are about the same size as RNA and DNA aptamers. This section will focus on antibody-siRNA conjugates or antibody-siRNA-NP therapies that enable targeting of tumor cells* in vitro* and* in vivo *(Figure 5).

#### 3.3.1. Antibody-siRNA Conjugates

Unlike aptamers and siRNA, which are similarly negatively charged, there have been concerns about highly negatively charged siRNA interfering with targeting of the antibody modified with siRNA or with cationic polymers. Cuellar and colleagues examined this potential problem and found that the siRNA did not interfere with the binding affinity of the antibody [88]. In this report, seven monomeric antibody-siRNA conjugates that differed in their chemical linkage or utilized several routes of internalization were examined. Although the conjugates demonstrated both targeting and silencing with cells and tumors* in vivo*, the silencing was at best modest (about 50%* in vitro* and 33% in the perivascular area of the tumor* in vivo*). No* in vivo *tumor efficacy studies were reported. The authors concluded that the two major problems for this approach were that the antibody conjugates clustered near blood vessels within tumors and that escape from the endosomes of tumors was limited. Notably, single-chain variable fragment antibodies were not tested in this study, which likely would improve tumor penetration and gene silencing with this approach.

Most tumor efficacy studies have utilized a fusion antibody-cationic peptide that is mixed with siRNA. Yao and colleagues complexed si*PLK1* through ionic interactions with a single-chain variable fragment Her2 antibody-protamine fusion protein. Each fusion fragment-protamine bound to approximately 6 siRNA molecules (Figure 5(a)). With the fusion siRNA construct administered biweekly, there was marked inhibition of Her2^+^ tumors (80 to 90%), whereas there was no effect of the therapy on the Her2^−^ tumors [89].* PLK1* mRNA in Her2^+^ tumors was knocked down by about 75% in the siRNA treatment group. In addition to binding siRNA, the cationic protamine likely has a role in lysis of endosomes, releasing siRNA into the cytosol. With a similar strategy, an EGFR antibody-si*Kras* complex reduced its target* in vitro* and* in vivo*, resulting in inhibition of the tumors by 75% compared to controls [90]. There have been concerns that these fusion protein-siRNA therapies, which are dependent on electrostatic interactions, might be too heterogeneous for clinical trials [88]. Although more biophysical characterizations are certainly required to understand and minimize the heterogeneity of antibody-siRNA conjugates, the same criticism could be leveled at polyplexes which have a degree of heterogeneity.

Although the TfR is upregulated in many tumors, investigators have also taken advantage of its presence on the blood-brain barrier. For example, Wang and colleagues used a transferrin antibody-attached to a NP to enable its transport across the blood-brain barrier [91]. In this orthotopic glioblastoma model, treatment with a transferrin scFv NP carrier of shsurvivin prolonged survival of the mice compared to those in the control treated group (*p* < 0.01) [91]. The antibody targeting transferrin not only augmented uptake by the tumor based on* in vitro* data but also increased transport of the antibody-shRNA complex through the blood-brain barrier. By using streptavidin-biotin linkage between the antibody and siRNA, Xia et al. also demonstrated the utility of a transferrin antibody to deliver siRNA targeting luciferase to orthotopic brain tumors [92]. Luciferase activity was reduced by 70–80% in the treated brain tumors.

While cationic peptides have been fused at the C-terminal end of single-chain antibody fragments through genetic engineering [89], a more flexible site-specific approach has been developed by Lu et al. [93]. After genetically incorporating an unnatural amino acid (p-acetyl L-phenylalanine) at different locations within 2 full-length and 1 Fab fragment antibodies, a cationic polymer was conjugated to this amino acid via oxime bonds. Two of the siRNA conjugates were effectively internalized by the Her2^+^ cells* in vitro* and consequently silenced their target gene by more than 70%. The reduced silencing by one conjugate was attributed to the proximity of the basic polymer and the antigen binding sites, interfering with cellular uptake. Thus, this method can be utilized with different types of antibodies, and antibody conjugates may be peptides or nonpeptide polymers.

#### 3.3.2. Antibody-siRNA-Nanoparticles

Antibodies, including the smaller molecular Fab and single-chain fragment variable derivatives, have been conjugated to liposomes and polymers to deliver chemotherapeutic agents, plasmids, and siRNA to tumors (Figure 5(b)) [94–104]. Conjugation of antibodies to NPs has been plagued by reduced binding affinity, heterogeneity of binding, and inadequate density, and consequently, a number of groups have investigated these issues to advance development of these targeted NPs. For instance, Deng and colleagues investigated whether different linkages of the EGFR2 Fab to PEG would affect silencing activity of the NP [6]. The PEG maleimide-derivative linker enhanced the uptake and silencing activity of the NP compared to the PEG-CO_2_H derivative. It is likely that the amide bond resulted in a PEG-CONH-Fab conjugate which interfered with the binding affinity of the antibody. Similar to what was previously discussed with the cyclodextrin nanoparticle [62], this illustrates that the method to form a nanoparticle is critical in developing the most effective silencing particle.

Dou and colleagues targeted Her2^+^ xenografts but with a scFv-Ab NP in which the siRNA targeted Polo-like kinase-1* (PLK1)* [95]. The core of the NP was composed of a PEG-poly(D,L-lactide) copolymer, a cationic lipid, and siRNA. The targeted NP augmented uptake significantly in Her2^+^ xenografts, had greater silencing of PLK1, and reduced tumor size to a greater extent than did untargeted NPs. The tumor efficacy of the targeted NP was particularly marked at lower dosages of therapy, underscoring the need for multiple dosages in these studies. In Her2^−^ tumors, there was little difference in the silencing and antitumor activity of targeted and nontargeting NPs. Combination of chemotherapy agents with siRNA has also been examined with targeted therapy. Zhao et al. incorporated docetaxel and si*PLK1* within a Herceptin Ab-labeled micelle [105]. The targeted micelle decreased the IC_50_ by 94.7% in highly expressing Her2^+^ cells compared to untargeted micelles.

By taking advantage of the overexpression of TfR on the blood-brain barrier, Partridge downregulated EGFR by 90% and prolonged survival of mice with orthotopic tumors treated with TfR-Ab-pegylated liposomes-sh*EGFR* [106]. An alternative strategy for treatment of brain tumors was based on their overexpression of the GD2 ganglioside. Shen et al. targeted neuroblastomas with a GD2 scFv-Ab theranostic iron oxide NP [101]. The GD2 scFv-Ab-NP containing si*Bcl-2 *reduced its target by about 60% and reduced the size of the neuroblastomas by about 50% compared to the nontargeting NP. Further corroboration was provided by MR imaging of the tumors which showed a 35% decrease in the T2 signal intensity in mice treated with the targeted NP compared to the nontargeted NP.

In pancreatic cells and cancers, there is an increased expression of CD44 transmembrane glycoproteins. As a result, Zeng et al. tested whether an NP consisting of CD44 scFV-Ab-PEG-polylysine copolymer in complex with si*KRAS* would inhibit pancreatic cells and tumors [94]. Although proliferation of a pancreatic cancer cell line (PANC-1) was somewhat inhibited with targeted therapy (versus nontargeted therapy), the number of colonies formed in soft agar was markedly reduced with cells treated with the targeted therapy. With imaging of the tumor and various tissues* in vivo*, there was an increased uptake of targeted NPs within implanted pancreatic tumors at several time points compared to that of untargeted NPs (*p* < 0.05). Uptake differences were further confirmed by confocal microscopy, which also demonstrated intracellular localization of the NP. In contrast, there was no difference between targeted and nontargeted therapy in their uptake in normal tissues. Despite the uptake differences, the differences in the tumor sizes were modest between groups of mice treated with the targeted and nontargeted KRAS NP. Lower dosages of the NP may have provided greater distinction between targeted and nontargeted NPs, as observed by Dou et al. [95].

### 3.4. Small Molecule Ligands

#### 3.4.1. Folate

The most common small molecule ligand that has been conjugated to NPs to target tumors is clearly folate. Folate has been attached to diverse groups of siRNA carriers including liposomes [107], an array of polymers [108–111], polymer-liposome [112] or polymer-micelle combinations [113], nanogels [114], packaging RNA [115], PEG conjugates [116], iron oxide NPs [117], mesoporous silica particles [118], and DNA tetrahedral structures [119]. Folate can bind with high affinity to its receptor, which is overexpressed on numerous solid tumors such as ovarian carcinomas, glioblastomas, and lung adenocarcinomas. Once the folate receptor binds with the folate-containing NP, the NP is rapidly internalized via receptor-mediated endocytosis, releasing its therapeutic content.

With so many publications on conjugation of folate to NPs, we have selected a few as representatives of the field. Li et al. demonstrated that folate coupled to cyclodextrin-PEI core in complex with si*VEGF* reduced the target mRNA and tumor size of HeLa xenografts by about 50 and 40%, respectively, compared to the nontargeted carrier [109]. In nasopharyngeal cancer xenografts, folate-linked lipid-based carriers of si*Her-2 *(three injections of 10 *μ*g of siRNA) reduced tumor growth significantly (approximately 35% decrease; *p* < 0.01), whereas the nonligand carrier reduced tumor size by less than 10% and was not significant [107]. With a tetrahedral DNA-based carrier, Lee and colleagues demonstrated that at least three folates per carrier were essential for optimal silencing with siRNA* in vitro*. In addition, maximal silencing was achieved when the folate ligands were in close proximity with one another on the tetrahedral structure. Moreover, this DNA-based carrier of siRNA injected intravenously had increased accumulation within the tumor (compared to other organs) and effectively reduced luciferase expression (about 60%) of human KB nasopharyngeal xenografts* in vivo*, although a nonfolate carrier was not compared [119]. Recently, Wagner's group used methotrexate-labeled NPs to target folate receptors on a KB cervical tumor model [120]. With the dual-functioning methotrexate not only targeting but also inhibiting tumor growth, the sequence-defined NP carrying an siRNA directed toward the oncogene, eglin 5 (EG5), markedly prolonged the survival of KB-bearing mice. The combination of methotrexate and si*EG5* incorporated in the NP (methotrexate/si*EG5* NP) resulted in the survival of 50% of the tumor-bearing mice for more than 70 days (with no recurrence of the tumor), whereas untargeted siEGF NPs or the folate/siEG5 resulted in all mice euthanized by day 40 (mice euthanized when tumor reached 1000 mm^3^ in size).

Some normal tissues such as lung and kidney in humans express high levels of folate receptors (primarily the alpha receptor), but these receptors are localized on the apical surfaces of highly polarized epithelial cells [121, 122]. As a result, the folate receptors on normal cells may not be accessible to blood-borne NPs. Indeed, little accumulation in the lungs of humans was observed with a folate-conjugate injected intravenously, indicating that off-target effects by folate-labeled NPs injected intravenously may be minimal. As a result, despite the high expression levels of receptors in lungs of humans compared to those of mice, their apical location likely minimizes differences in biodistribution of intravenously delivered folate-NPs between mice and men [122].

#### 3.4.2. Anisamide

Sigma receptors, overexpressed on the membranes of several malignant tumors and dividing normal cells, bind tightly to haloperidol and analogues, including anisamide. Interestingly, binding of these exogenous ligands to the sigma receptors and in particular the sigma-2 receptor resulted in death of malignant cells (but not normal cells) by apoptotic and nonapoptotic mechanisms. Nevertheless, targeting the sigma receptor of tumors with siRNA NPs enabled their uptake and enhanced their antitumor activity. For example, Huang' s group has published several papers demonstrating that anisamide-containing lipid-based NPs are significantly better carriers of pooled siRNA (targeting* VEGF*,* MDM2*, and* c-myc*) than are the nontargeted NP in different models of lung cancer [123–125]. More recently, combination therapy utilizing photodynamic therapy and anisamide-containing lipid-based carriers of si*HIF-1α* substantially inhibited the growth of squamous cell carcinomas in mice compared to either therapy alone [126]. In addition, Guo et al. demonstrated that the anisamide-cyclodextrin carrier of si*VEGF* was about 35% and 60% more effective than the nontargeted and PBS-treated groups, respectively, in reducing the size of prostate cancer xenografts [127]. Thus, in a diverse group of tumors, targeting the sigma receptor increased delivery of the siRNA-NP and holds future promise.

#### 3.4.3. Galactose

NPs containing galactose have been developed to deliver therapeutic agents (chemotherapeutic agents, DNA) to the asialoglycoprotein receptor on liver cells for over 30 years [128, 129]. Han and colleagues revisited and exploited this targeting ligand by developing galactose-containing mesoporous silica NPs, enabling their entry into hepatocarcinoma cells. These systemically delivered NPs sequentially released si*VEGF* and then doxorubicin into hepatocarcinoma xenografts implanted into nude mice. Compared to controls, there was a 90% reduction in tumor size [130]. Without galactose attached to the surface of the NP, the efficacy of NPs was reduced by about 20%. Han and colleagues also used the oral route to deliver galactose-containing NPs [131]. Specific amounts of galactose conjugated to trimethyl-chitosan markedly enhanced uptake of the siRNA and shRNA by the tumor xenografts. Whereas galactose was important for the uptake and enhanced specificity of the NP, the trimethyl-chitosan was essential for its transport through the intestinal wall. Interestingly, NPs carrying si*VEGF* and shsurvivin were synergistic in antitumor activity. In both studies, the liver, which expresses elevated amounts of the asialoglycoprotein receptor, did not show toxicity from these NPs. Although these studies show promise, further studies are required to demonstrate that the liver is not a major target of galactose ligand NPs.

### 3.5. Peptide Ligands Targeting Tumors

#### 3.5.1. T7 Peptide (HAIYPRH)

The T7 peptide was isolated by a phage display approach using cells that express high levels of the TfR [132]. Unlike most peptide ligands, the T7 peptide was reported to have a high affinity (Kd about 10 nM) for its receptor [133] and since it binds to a different site of the receptor than does transferrin, the endogenous ligand does not inhibit uptake of T7-mediated receptor-endocytosis [132, 133]. Although the vast majority of NPs have targeted TfR with transferrin or an antibody, Gao and colleagues targeted the receptor with the T7 peptide [134]. The si*EGFR2* NP labeled with the T7 peptide inhibited tumor size about 40% more than did the untargeted NPs, and 70% more than the glucose-treated group. The tumor-inhibitory results of the various treatment groups correlated with degree of downregulation of the EGRF2 protein. Similar to the transferrin ligand which coated the surface of cyclodextrin NP [5], T7 did not appear to increase accumulation of the NP within the tumor. Notably, there was no evidence of toxicity or cytokine induction with the T7 targeted particles.

#### 3.5.2. MMP2-Cleavable Octapeptide (GPLGIAGQ)

**Figure 6:**
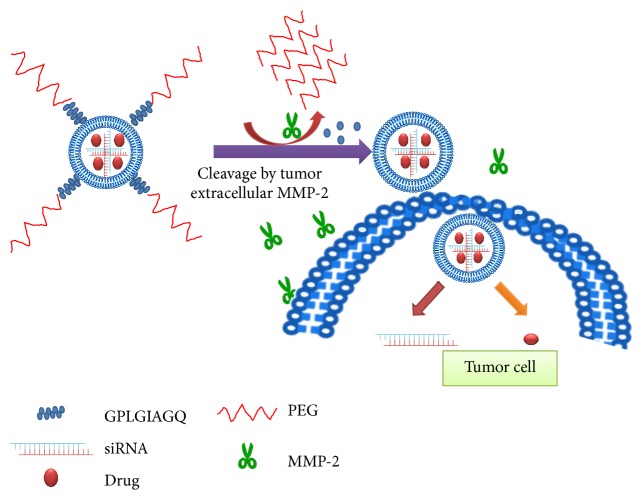
Enhanced tumor uptake of MMP2-degraded NP. Several tumors secrete high levels of the MMP2 enzyme into their stroma. By incorporating the substrate, GPLGIAGQ, between PEG and the NP, the MMP2 cleaved the peptide and releases PEG from the NP. This enables the NP to bind to the negatively charged surface of tumor cells with subsequent endocytosis of the NP.

This cleavable peptide linker between PEG layer and the remainder of the NP represents a unique method of tumor targeting (Figure 6) [135]. Unlike the vast majority of NPs that target the tumor cells or their stroma, the uptake of these enzyme-sensitive NPs in tumor cells is greatly enhanced by metalloproteinase-2 (MMP2), an enzyme secreted at high levels of many tumors, but found in low levels in most normal tissues. When the peptide substrate (GPLGIAGQ) incorporated on the surface of the NP is cleaved by MMP2, the PEG shield is released from the NP, enabling the multifunctional micelle particle containing siRNA and paclitaxel to interact with tumor cell surface and be imported into the cell. This targeting strategy was validated with a nonsmall cell lung cancer (A549)* in vitro* and* in vivo*. Paclitaxel and the siRNA were codelivered with the MMP2-sensitive NP to more than 98% of the cells* in vitro*, whereas they were codelivered to about 70% of cells with the MMP2-insensitive NP. Similarly, the MMP2 showed improved codelivery of siRNA and paclitaxel to tumor cells* in vivo* (14.4%, sensitive versus 6% uptake, insensitive).

#### 3.5.3. CP15 (VHLGYAT)

The CP15 peptide, identified with a phage display library, is able to target colon cancer cells yet it is not able to recognize normal intestinal epithelial cells. The C15 peptide (VHLGYAT) was conjugated to the PEG-chitosan (CS) copolymer prior to addition of a cross-linker (sodium tripolyphosphate) and siRNA. Silencing of the targeted PLK1 mRNA and protein in human tumor xenografts with the C15-PEG-CS NPs was approximately 50% of that in the control siRNA group [136]. Although silencing differences between the targeted C15-PEG-CS NP and untargeted PEG-CS NP groups were not compared, accumulation in the tumor was modestly greater (about 20%) with the targeted NP compared to the untargeted NP.

#### 3.5.4. FSH (FSH-*β*, 33–53 Amino Acids, YTRDLVYKDPARPKIQKTCTF); LHRH (QHTSYkcLRP)

Both FSH and LH ligands bind receptors present on the normal ovary as well as on ovarian cancer cells, and peptide fragments of these hormone ligands have been used to target NPs. Hong and colleagues examined the efficacy of NPs composed of FSH*β* (33–55)-PEG-PEI and the cytokine si*Gro-α* against ovarian tumor cells [137]. Compared to the untargeted NP in complex to si*Gro-α*, the numbers of cells and migration distance of cells were reduced by about 35% and 75%, respectively. Although these studies with siRNA were* in vitro*, the same laboratory had previously demonstrated that tumor volume and weight were significantly reduced with the FSH-targeting ligand NPs containing paclitaxel compared to the nontargeting NPs [138].

Similarly, Shah et al. targeted ovarian cancer with a LHRH peptide-polypropylenimine-NP containing paclitaxel and a siRNA that downregulates CD44 [139]. The LHRH sequence, QHTSYkcLRP, was conjugated to PEG through interaction of its thiol group with the maleimide on the PEG. In an* in vivo* experiment, the LHRH-peptide-NP-Pax/si*CD44* prevented growth and indeed caused regression of the ovarian tumor xenografts that were about 400 mm^3^ prior to initiation of therapy by about 50% (after the 8th treatment). The targeted NP containing paclitaxel or si*CD44* reduced tumor growth markedly more than did the untargeted NP groups. Moreover, the CD44 protein in the xenografts was clearly downregulated by the siRNA when the LHRH-NP-paclitaxel and the LHRH-NP-paclitaxel/si*CD44* groups were compared.

#### 3.5.5. Gastrin-Releasing Peptides (GRPs) (CGGNHWAVGHLM)

The gastrin-releasing peptide, isolated from the peptide phage library, binds to the BB2 receptor on several malignancies including colon, breast, lung, and prostate cancer cell lines. The authors found that the GRP peptide did bind to the MDA-MB-231 cells but did not bind to endothelial cells [140]. Moreover, the cells treated with GRP-siSurvivin conjugates had survivin mRNA levels that were about 15% of the GRP-scrambled siRNA or siRNA controls.

#### 3.5.6. RVG-Brain Delivery (YTIWMPENPRPGTPCDIFTNSRGKRASNG)

Similar to the transferrin-mediated NPs, the rabies virus glycoprotein- (RVG-) derived peptides may augment transport of NPs across the blood-brain barrier to the central nervous system [141]. To date, there has been only one report (an* in vitro* study) examining RVG-directed NPs with siRNA targeting cancer [142]. That the acetylcholine receptor for the RVG peptide is found on the surface of glioblastoma cells is indicated by the enhanced uptake of RVD-NPs in U87 cells compared to untargeted NPs. In contrast, there was no difference in the uptake of RVD-NPs and untargeted NPs in the cervical HeLa cell line. Downregulation of GADPH mRNA was about 25% more with the targeted* siGADPH* NP than with the untargeted NP.

## 4. Conclusion

Diverse numbers of ligands and approaches to augment siRNA delivery to tumor cells and to tumors in mouse models have been described in this review. More complex ligand targeting NPs, no matter how promising in preclinical trials, must be justified economically and overcome barriers with production scale-up for commercial manufacturing. It is thus not surprising that the less complex ligands (i.e., antibodies and aptamers) are investigated initially in clinical trials. For example, aptamers are being tested in clinical trials and CenR peptides with their recent demonstration of preclinical efficacy may not be far behind. In addition, antibodies directed against Her-2/neu or EGFR (Her-1) tumor-associated receptors or antigens have already demonstrated marked clinical efficacy. Nonetheless, the progressive shift to the use of mAb as targeting ligands for toxins or chemotherapeutics certainly supports development of ligand-targeted forms of siRNA.

Of course, the economically more feasible targeted siRNA therapies (antibody-siRNA or aptamer-siRNA conjugates) could be less effective in some cases than more complex NPs. Multicomponent NPs may have significant advantages due to greater tumor specificity (i.e., multivalency), reduced need to use chemically modified siRNA, and enhanced intracellular siRNA delivery avoiding lysosomal degradation. Nevertheless, because of the challenges with their manufacturing, the more complex multicomponent NPs may not be tested in patients despite their preclinical efficacy. This remains a major challenge in the field for the most promising multicomponent NPs to be tested for clinical efficacy.

The type of ligand to conjugate on the NP may also be challenging. This is illustrated by the number of approaches to identify a ligand targeting the TfR (Table 3). Investigators have used the natural ligand, monoclonal antibodies, single-chain fragment antibodies, aptamers, and even peptides to target the TfR. Which of these strategies is preferred will likely be based on their availability, ease of synthesis and purification, their target affinity, and selectivity as well as the size of the NP. Of these approaches for TfR, the aptamer and the peptide ligand are particularly attractive because of their high affinity for their target, their low molecular weight, and relative ease of synthesis. The greater the size of the NP, the more concern there is about the molecular weight of the ligand. Thus, given a choice between conjugating an antibody or scFv antibody or aptamer to a liposome, it would seem that lower molecular weight ligands would enable greater penetration of the NP within the tumor. Moreover, the peptide ligand differs from the other ligands for TfR in that its binding site is different from the site for transferrin; as a result, the uptake of the T7 NP will not be affected by the relatively high serum levels of endogenous transferrin (25 *μ*M).

An additional layer of complexity is the interaction between the ligand-siRNA therapeutic and the blood components. Although considerable attention has been given to measuring the biophysical parameters of the NP, less has been given to what may occur to the NP* in vivo*. Silencing experiments done in the presence of medium plus serum reduced the targeting ability of the transferrin-linked NP, by enabling a corona of proteins to surround the NP [57]. This finding will likely extend to other ligands in addition to transferrin, and the presence of the corona may depend on the core particle (i.e., cationic versus neutral liposomes) and the type of ligand (aptamer, endogenous ligand, antibody, small molecule, and others).

In addition to the many promising ligands that have been investigated for targeting siRNA to tumors, many other ligands that have considerable potential have not been tested with siRNA. One example is the urokinase plasminogen activator (uPA) ligand for its receptor, which is upregulated in the majority of pancreatic (about 90%) and breast cancers (60–90%). With a recombinant N-terminal peptide of the uPA, high levels of the NP injected via the tail vein were found in tumors with low levels in normal tissue [143]. Indeed, the number of known ligands that need to be tested and compared* in vitro* and/or* in vivo* for enhanced siRNA efficacy against tumors is certainly challenging. Nevertheless, we think it is likely that the many permutations of the ligand, siRNA, and NPs will yield promising drug candidates for improved treatment of a wide range of life-threatening cancers.

## Figures and Tables

**Table 1:** Ligands targeting endothelial and tumor cells.

Ligand/target^1^	Carrier	siRNA and/or active moiety	Cells	Results	Other comments	References
cRGD^2^/*α*v*β*3/*β*5	Polymer (PEI)^3^	si*VEGFR2*	N2A neuroblastoma allografts, implanted SC	Marked inhibition of tumor growth (versus nontargeted NP)	May target physiologic angiogenesis	[22]
	Blocked copolymers micelles	si*VEGF*/si*VEGFR2*	HeLa xenografts, SC	Marked inhibition of tumor growth	Silencing both *VEGFR2*/*VEGF* more effective than either alone	[27]

APRPG/VEGFR-1	Polymer (PEI)	si*VEGF*	MCF7 xenografts, SC	Targeted and nontargeted PEG-NP inhibited tumor similarly	Reduction of intratumoral VEGF and mRNA greater with targeted NP	[34]
	Liposome	si-*mTOR*	B16F10, IV, tumor-laden lungs	Targeted and nontargeted NP reduced tumor burden similarly	Wild-type PTEN expressing tumors	[35]

cNGR/CD13	Liposome	si*c-MYC*/doxorubicin	HT-1080 (fibrosarcoma) xenografts, SC	Tumor uptake and inhibition greater with targeted NP	HT-1080, a CD13+ cell	[38]

F3/nucleolin	Silk-polylysine-copolymer NP	Plasmid-based luciferase expression	MDA-MB-231 xenografts, SC	Targeted NP increased gene expression in tumors	Only study *in vivo* with F3 ligand; F3 more effective than CGKRK *in vitro*	[44]
	Liposomes	si*PLK1*	PC3 (in vitro study)	Uptake and silencing greater with targeted therapy	Cells pretreated with si*PLK1* were more sensitive to the inhibitory effects of paclitaxel	[42]

CGKRK/p32	Nanoworms	Mitochondria-targeting peptide	Orthotopic breast cancer model (MCF10CA1a)	Targeted NP gave greater tumor inhibition than nontargeted NP	Addition of iRGD increased tumor inhibition of targeted NP	[45]

iRGD	PEI micelles	Shsurvivin/paclitaxel	A549 lung cancer xenografts, SC	Greater tumor size inhibition with targeted therapy	Plasmid-based expression of shRNA; dose-response inhibition; no siRNA studies yet	[51]

^1^Selected representatives of targeted nanoparticles. ^2^See text for amino acid sequences of ligands; ^3^PEI, polyethylenimine; NP, nanoparticle; siVEGFR2, small inhibitory RNA targeting VEGFR2; PLK1, polo-like kinase 1; SC, subcutaneous; shRNA, small hairpin RNA expressed by plasmids; siRNA, small inhibitory RNA.

**Table 2:** Ligands targeting tumor cells.

Ligand/target^1^	Carrier	siRNA and/or active moiety	Cells	Results	Other comments	References
*High MW endogenous ligands*						

Transferrin (Tfr)/TfR receptor	Polymer (cyclodextrin)	si*RRM2*	Neuro2A, murine neuroblastoma, implanted SC	Greater tumor inhibition with targeted than nontargeted NP	Dose-related efficacy using transferrin ligand	[59]

Hyaluronic acid/CD44	Liposomes	si*PLK1*	U87MG, human glioblastoma, orthotopic	Prolonged survival, reduced PLK1 mRNA by 80% in tumor xenografts versus control siRNA	CD44 increased on cell surfaces of many cancers	[63]

ApoA1/SR-B1	Liposomes	si*VEGF*	MCF-7, SC	Inhibited tumor size and prolonged survival	Hepatocellular carcinomas have been targeted	[72]

*Aptamers*						

Aptamer/PSMA^2^	Aptamer-siRNA chimera	si*PLK1*	PSMA^+^ (22Rv) versus PSMA^−^ (PC-3) cells, SC	Tumor regression of 22Rv xenografts treated with apta-mer-PLK1 siRNA	PSMA upregulated in prostate cancer	[78]
Aptamer/PSMA	Aptamer- dsRNA binding domain- polyhistidine chimera	si*GFP*	PSMA^+^ (LNCaP) versus PSMA^−^ (PC-3) cells, *in vitro* study only	Silenced GFP efficiently in LNCaP cells	dsRNA-binding domain binds to siRNA; chimera may not efficiently lyse endosomes *in vivo*	[80]

Aptamer/PSMA	Polymer (atelocollagen)	(miR15a and miR-16-1)	Bone metastatic prostate model-LNCap cells	Prolonged mouse survival versus nontargeted NP	Reduced expression of Bcl-2, cyclin D1, and Wnt3a *in vitro* with targeted NP	[86]

*Antibodies*						

scFv/Her2^+^	scFv-protamine-conjugate	si*PLK1*	Her2^+^ versus Her2^−^ breast cancer, orthotopic	Marked tumor inhibition on Her2^+^ tumors *in vivo* versus no/little effect on Her2^−^ tumors	Ionic interaction between siRNA and protamine; siRNA cocktail targeting *PLK1*, *CCND1*, and *AKT* more effective than PLK1 alone	[89]

scFv/Her2^+^	Polymer (PLA)	si*PLK1*	Her2^+^ (BT474) versus Her 2^−^ (MCF7) breast cancer, orthotopic	Targeted NP inhibited both Her2^+/-^ xenografts but showed the most efficacy toward Her2^+^ tumors	Dose-dependent antitumor response of the targeted NP was observed	[95]
*Small molecule nonpeptide ligands*						

Folate/folate receptor	Low MW PEI cross-linked with by cyclodextrin	si*VEGF*	HeLa xenografts, SC	Targeted NP reduced tumor size more than nontargeted	Folate receptor is widely distributed in epithelial cancers	[109]
	Tetrahedral DNA	si*GFP* siLuciferase	KB expressing GFP *(in vitro)* or luciferase, SC	Folate NP, given IV or IT, reduced tumor luciferase expression by 60%	3 ligands on same face of tetrahedron to maximize uptake; well-defined NPs	[119]
	Polymer (oligoamine amides)	si*EG5*	KB xenografts, SC	Methotrexate/siEG5 NP enhanced survival	Methotrexate targets folate receptor, small NPs (~6 nm) delivered IT	[120]

Anisamide/sigma-1, -2 receptors	Liposome	si*MDM2*, sic-*MYC*, and si*VEGF*	B16F10 murine melanoma, SC, and lung metastases	Reduced primary and metastatic tumor burden and prolonged survival	Receptor widely distributed in tumors and proliferating cells; sigma-2 ligands induce apoptosis in tumors	[125, 144]

Galactose/asialoglycoprotein receptors	Multilayered NP (mesoporous silica NP, polymers)	si*VEGF*/doxorubicin	QGY-7703, a hepatocarcinoma, SC	Marked inhibition with targeted NP (91.3%) versus nontargeted NP (75.2%)	Ligand targets primarily liver cells	[130]

*Peptide ligands*						

T7^3^/transferrin receptor	Cholesterol-grafted polymer/lipid hybrid	si*EGFR*	MC7 xenografts, SC	About 50% greater reduction in tumor size with targeted than nontargeted NPs	Binding of T7 to receptor is independent of endogenous transferrin concentration	[134]

MMP2-cleavable peptide	PEI-lipid hybrid micelle	Fluorescently-labeled siRNA and paclitaxel	A549 lung cancer xenografts, SC	Enhanced tumor uptake by 2.4-fold with MMP2-micelle	MMP2-triggered PEG deshielding	[135]

CP15/unknown	Polymer (chitosan)	si*PLK1, *biotin-labeled RNA	SW480 colon cancer cells, SC	Modest increase in tumor uptake/specificity of targeted NP (versus nontargeted NP)	Identified by phage display; primarily targets colon cancer; study requires validation	[136]

LHRH/LHRH receptor	Polymer (PPI)	si*CD44*/paclitaxel	Ovarian cancer xenograft, SC	Targeted therapy significantly more effective than nontargeted therapy	Combination of paclitaxel and siCD44 NP most effective in reducing tumor size	[139]

GRP/BB2 receptor	Conjugate	Fluorescently-labeled siRNA, siSurvivin	MDA-MB-231 xenografts *(in vitro)*	Competitive inhibition indicates specific uptake of GRP-siRNA conjugates into cells; marked reduction of survivin with targeted NP	Requires *in vivo* validation; BB2 reported to be on breast, prostate, lung, and colon cancers	[140]

RVG/acetylcholine receptor	Polymer (cyclodextrin)	si*GADPH*	U87 glioblastoma, HeLa (RVG- control), *(in vitro)*	Targeted NP reduced GADPH mRNA about 25% more than nontargeted NP in U87	Requires *in vivo* validation; *in vitro* efficacy is modest	[142]

^1^Selected representatives of targeted nanoparticles. ^2^PSMA, prostate specific membrane antigen; scFv, single-chain variable fragment antibody; MMP2, metalloproteinase-2; LHRH, luteinizing-hormone releasing hormone; GRP, gastrin-releasing peptide; BB2, a subtype of the bombesin receptor; RVG, rabies virus glycoprotein; dsRNA, double-stranded RNA binding domain; PLA, poly(D,L-lactide); PEI, polyethylenimine; PPI, polypropylenimine; RRM2, ribonucleoside-diphosphate reductase subunit M2; PLK1, polo-like kinase-1; EG5, eglin 5; GFP, green fluorescent protein; EGFR, epidermal growth factor receptor; GADPH, glyceraldehyde-3 phosphate dehydrogenase; SC, subcutaneous; NP, nanoparticle; IT, intratumoral. ^3^See text for amino acid sequences of ligands.

**Table 3:** Multiple strategies for a single target.

Target	Ligand	Carrier	siRNA target	Cell targets	Results of targeted ligand	Reference
Transferrin receptor	Transferrin	Liposome	*RRM2*	MV4-11, an AML line	Improved tumor accumulation (versus nontargeted NP) and enhanced silencing (versus control siRNA NP)	[54]
	T7 peptide	Liposome-polymer hybrid	*EGFR*	MCF-7	Marked tumor size reduction (versus nontargeted NP)	[134]
	Aptamer	SNALP liposome	*GFP or LamA/C*	HeLa-EGFP cells *(in vitro)*	Enhanced uptake and silencing (versus transferrin-NP)	[84]
	scFv^1^	scFv-polylysine conjugate	Survivin	U87 glioma cells	Enhanced silencing and improved survival of mice with orthotopic tumors (versus nontargeted NP)	[91]
	scFv	Liposome	(Fluorescently-labeled siRNA)^3^	Pancreatic, prostate, and melanoma cell lines	Specific delivery to orthotopic and metastatic tumors *in vivo*	[145]

CD44	HA	Liposome	*PLK1*	U87MG glioblastoma cells	Decrease *PLK1* by 80%; prolonged survival in orthotopic model	[63]
	scFv	Polymer	*KRAS*	PANC-1, pancreatic cancer	Enhanced *in vitro*/*in vivo* silencing and efficacy (versus nontargeted NP)	[94]
	scFv	Polymer- (PEI-) iron oxide	Nontargeting siRNA	High (SGC-7901) versus low (A375) CD44 expressing cells	Increased uptake of targeted NP via magnetic resonance in SCC-7901 compared to A375	[146]
	Aptamer	Liposome	—	CD44^+^ (AS49, MDA-MB-231) versus CD44^−^ (NIH/3T3) *(in vitro)*	Increased accumulation of targeted-NP in CD44^+^ cells	[147]

p32	Peptide (LyP-1)	Polymeric micelles	*ID4*	OVCAR-4, 8	Marked tumor reduction *in vivo*, administered IP	[10]
	scFv, trimerbody	—	—	MDA-MB-231	Widespread distribution of labeled antibody in tumor xenografts, particularly with the trimerbody	[148]

VEGFR2	VRBP1 peptide^2^	—	—	HUVEC, H460	VRBP1 reduced size of tumor xenografts	[149]
	Aptamer	Magnetic particle	—	PAEC, U87MG glioblastoma	Increased MRI signal in tumors *in vivo* (versus nontargeted NP)	[150]

^1^scFv, single-chain variable fragment antibody; HA, hyaluronic acid; SNALP, stable nucleic acid lipid particles; PEI, polyethylenimine; RRM2, ribonucleoside-diphosphate reductase subunit M2; EGFR, epidermal growth factor receptor; GFP, green fluorescent protein; LamA/C, lamin A/C; PLK1, polo-like kinase 1; ID4, inhibitor of DNA binding 4; AML, acute myeloid cell leukemia; HUVEC, human umbilical vein endothelial cells; PAEC, porcine aortic endothelial cells; CPP, cell penetration peptide; NP, nanoparticle. ^2^Sequence of VRBP1 peptide - YDGNSFYEMVVGVKPASES; ^3^nontargeting siRNA.
